# Granulomatosis with Polyangiitis-Mimicking Advanced Gynecological Cancer: A Case Report and Systematic Review of the Literature

**DOI:** 10.3390/jpm12020289

**Published:** 2022-02-16

**Authors:** Augusto Pereira, Javier F. Magrina, Paul M. Magtibay, Beatriz G. Stamps, Elena Muñoz-Nuñez, Tirso Perez-Medina

**Affiliations:** 1 Department of Gynecologic Surgery, Puerta de Hierro University Hospital, 28222 Madrid, Spain; elenamn1994@gmail.com (E.M.-N.); tirsoperezmedina@gmail.com (T.P.-M.); 2 Department of Medical and Surgical Gynecology, Mayo Clinic Hospital, Phoenix, AZ 85054, USA; jmagrina@mayo.edu (J.F.M.); magtibay.paul@mayo.edu (P.M.M.); stamps.beatriz@mayo.edu (B.G.S.)

**Keywords:** granulomatosis with polyangiitis, vagina, female genital tract, vasculitis, Wegener granulomatosis

## Abstract

(1) Background: Granulomatosis with polyangiitis (GPA) is a necrotizing vasculitis that mimics gynecologic cancer. In GPA patients, the genitourinary system is affected in <1%. The objective of the study was to provide a systematic review of the literature of GPA patients with gynecological involvement. (2) Methods: PubMed and Embase were searched from inception to July 2021 for GPA patients with gynecological involvement Medical Subject Headings (MeSH) and free-text terms. Exclusion criteria were other language, review articles, pregnancy, fertility, or male patients. Data were extracted on clinical evolution, symptoms, examinations findings, diagnosis delay, treatment, outcome, patient status, and follow-up. (3) Results: Seventeen studies included data from patients with GPA and primary or relapsed gynecological involvement. 68% of the authors of this review thought the patient had cancer. The main gynecological symptom is bleeding, but exclusive gynecologic symptomatology is rare (ENT: 63%, lungs: 44%, kidneys-urinary tract: 53%). GPA could affect all areas of the genital tract, but the most frequent location is the uterine cervix. Medical treatment for GPA is effective. (4) Conclusions: GPA of the female genital tract must be considered when biopsies of an ulcerated malignant-appearing cervical or vaginal mass are negative for malignancy even when they are unspecific. Rheumatology consultation is indicated.

## 1. Introduction

Since January 2011, the Board of Directors of the American College of Rheumatology, the American Society of Nephrology, and the European League Against Rheumatism recommended that the name “Wegener’s granulomatosis” be changed to “granulomatosis with polyangiitis” (GPA) [1].

The prevalence is estimated between 1/6400–42,000 worldwide with an annual incidence between 1/84,000–475,000 [2]. GPA is a rare antineutrophil cytoplasmic antibody (ANCA)-associated vasculitis characterized by necrotizing inflammation of vessels, resulting in tissue ischemia. GPA is a multiorgan disease. Ear, nose, and throat (ENT) symptoms are present in 50–95% of patients, bronchopulmonary symptoms in 60–80%, and renal disease in 60–80%. Other sites of involvement with a lower incidence are skin in 10–50% of patients, peripheral nerves in 25%, central nervous system in 10%, and ocular in 7–8%. Patients with genitourinary involvement, cervix, urethra, and vagina, are extremely rare, <1% of patients with GPA [3].

Diagnosis remains delayed in acute settings in spite of a better understanding of this disease. It is usually based on clinical findings, imaging studies, and biochemical tests, as well as on the detection of serum antineutrophil cytoplasmic autoantibody (ANCA)-associated vasculitis, mainly PR3-ANCA, a biopsy should be performed to confirm the diagnosis [4].

GPA is defined, according to the Chapel Hill criteria, as a necrotizing granulomatous inflammation that affects the respiratory tract, with necrotizing vasculitis of small and medium vessels (for example, capillaries, veins, arterioles, and arteries). The etiology is unknown, but it is accepted that the immune system is involved in this pathogenesis. In the vast majority of cases, the combination of suggestive clinical characteristics and the presence of cytoplasmic-staining ANCA may be sufficient. If the are diagnostic doubts, histologic confirmation such as lung biopsy is needed [5].

Typically, women presenting with a large cervical or vaginal mass and unilateral ureteral obstruction have a locally advanced gynecologic malignancy. The diagnosis is usually straightforward, with a simple biopsy and pathologic assessment of the mass, followed by a metastatic workup when a malignancy is confirmed. However, in rare cases, biopsies are negative for malignancy, resulting in a diagnostic dilemma requiring further investigation. We report a patient referred to our institution for a presumed advanced vaginal carcinoma. Her history was remarkable for a previous hysterectomy for benign disease more than 30 years prior. Imaging confirmed a left ureteral obstruction, and biopsies were negative for malignancy. A final diagnosis of GPA was established when the patient had a CR to medical management.

Our objective is to provide a systematic review of the literature of GPA patients with gynecological involvement and to emphasize that a specific tissue diagnosis of GPA is not necessary to implement medical treatment when other conditions are unlikely to be responsible for the presenting pathology.

### Case Report

A 75-year-old white woman presented to Mayo Clinic in Arizona with a 1-year history of pelvic pain, intermittent hematuria, vaginal bleeding, and an undiagnosed and untreated large pelvic mass infiltrating the upper vaginal half, obstructing the left ureter. Her history was important for a total abdominal hysterectomy and bilateral salpingo-oophorectomy 36 years earlier for fibroids. Her other past medical history was unremarkable. Biopsies of the vaginal mass performed elsewhere were reviewed and found to be granulation tissue with necrosis, negative for malignancy. A laparoscopy performed at an outside institution was non-diagnostic. The patient was monitored for more than a year by her local gynecologist without further workup or therapy despite worsening symptoms. A repeat laparoscopy was recommended when she elected to come to our institution for consultation.

General physical examination was negative other than an ulcerated, exophytic mass extending along the anterior vaginal wall from the vaginal cuff to the urethra-vesical junction. The mass was firm and fixed to the left pubic ramus. The clinical diagnosis was advanced vaginal carcinoma. Multiple superficial and deep biopsies were obtained and showed nonspecific acute and chronic inflammation with necrosis and granulomatous inflammation. No evidence was found of vasculitis or malignancy.

Computed tomography (CT) showed a 4-cm vaginal mass involving the vaginal cuff, bladder base, and obstruction of the left ureter with hydronephrosis. Cystoscopy revealed a bulging trigone from an extrinsic mass with a non-visualization of the left ureteral meatus. Bladder biopsies showed necrotizing granulomatous inflammation. A percutaneous, antegrade left ureteral stent was placed to drain the obstructed kidney.

Further histologic stains for acid-fast, fungal organisms and cytokeratin were negative. Tissue cultures were negative for fungus and actinomyces. Reference blood tests included c-ANCA test, serum fungal serologies; interferon g (QuantiFERON; Qiagen, Hilden, Germany); antinuclear antibody and extractable nuclear antigen antibody profiling; and C3, C4, total hemolytic complement, rapid plasma reagin, and cyclic citrullinated peptide antibodies. The sedimentation rate was 17 mm/hour, and C-reactive protein concentration was 6.9 mg/L. Head and chest CT scans were negative.

Findings did not confirm but raised the suspicion of GPA in the absence of other plausible conditions. Biopsies revealed granulomatous necrosis without polyangiitis. Blood test results were inconclusive. There was no history of GPA and absence of pulmonary or renal involvement. The patient was referred to the Division of Rheumatology, Allergy, and Clinical Immunology, and a therapeutic trial of 4 doses (375 mg/m^2^) of weekly intravenous rituximab was offered to the patient, affirming that GPA was the most likely albeit not confirmed diagnosis. After the 4-week trial, CT revealed a marked reduction in the vaginal mass. The patient underwent a change in treatment to an increasing weekly oral dose of methotrexate, to a maximum of 20 mg, and 1 mg of oral folic acid daily. At 6 months, repeat CT showed complete regression of the vaginal mass, a normal bladder, and normal ureters. Cystoscopy was also normal, and the ureteral stent was removed. Pelvic examination was negative other than vaginal adhesions at the site of previous vaginal involvement. The patient continues to be asymptomatic with no evidence of recurrence after 53 months of follow-up.

## 2. Materials and Methods

### 2.1. Search Strategy

A systematic literature review was undertaken. Search terms were used as free terms and as Medical Subject Headings (MeSH) or Emtree terms (indexed on Pubmed or Embase from inception to July 2021): (a) The following free terms were used on all databases: “granulomatosis with polyangiitis” OR “Wegener granulomatosis”, AND “cervix”, “uterus”, “vagina”, “vulva”, “ovary”, “adnexa”, “adnexal” OR “gynecologic disease”. The following MeSH terms were used: “granulomatosis with polyangiitis”, OR “Wegener granulomatosis”, AND, “cervix uteri”, “uterus”, “vagina”, “vulva”, “ovary”, “adnexa uteri”, “adnexal uteri”, “gynecologic disease, female”, “uterine hemorrhage”, “metrorrhagia”, “gynecologic neoplasm, female”, “pelvic neoplasm”, “gynecology”, “genital female, disease”, “female urogenital, disease”, OR “genital neoplasm, female”, “genitalia, female”. The following Emtree terms were used: “granulomatosis with polyangiitis” OR “Wegener’s granulomatosis”, AND, “gynecologic disease” “gynecology” OR “female genital tract tumor”. Hand-searching of citations was carried out on case series and reviewed studies to allow us to identify references that might have been missed in previous searches.

### 2.2. Selection Criteria

The databases searched were PubMed from 1957 to July 2021 and Embase from 2011 to July 2021. Articles were included if describing patients primarily located in the female genital tract, case series, English language, and had an abstract. Exclusion criteria were other language, review articles, pregnancy or fertility-related issues, or male patients.

### 2.3. Data Collection and Analysis

All articles were screened on the basis of title and abstract (AP, JFM). The following data were extracted: title, author, year, journal, number of cases, age, clinical evolution, the initial presentation of symptoms, ENT, urologic and gynecological symptoms, lung findings, examinations findings, diagnosis delay, type of treatment, outcome, time to CR or PR, first and second recurrence, interval to recurrence, patient status, and follow-up. All data were included in an EXCEL spreadsheet, and the final selection of the articles was downloaded for full review (AP, JFM); any disagreement was resolved by discussion.

All retrieved articles were case reports with or without literature review; due to the high heterogeneity of data obtained, a descriptive narrative review was planned instead of a meta-analysis. The risk of bias was minimized by adhering to the PRISMA statements [6]. In addition, we described the flow diagram of study search and systematic review with reasons for being excluded, removed or not retrieved, using PRISMA guidelines. All data included in the study (in total 19 patients from 17 studies) were compiled in 4 tables according to the patients with GPA and primary gynecological involvement, and gynecological relapse.

### 2.4. Definitions

In order to unify the criteria for our study, diagnosis of GPA was established based on pathological reports and/or c-ANCA test. Diagnosis delay was defined as the time from the initial presentation of symptoms to diagnosis. GPA treatment consists of an “initial induction phase” to achieve long-standing remission of the active disease, followed by a “maintenance phase” to extend remission and avoid recurrence. Among the GPA treatments described in the literature, glucocorticoids in combination with either rituximab, cyclophosphamide, methotrexate, or azathioprine have been proposed better than monotherapy with prednisone or methylprednisolone. Complete remission (CR) was defined as the absence of active disease, and partial remission was referred to as the persistence of signs and symptoms of active disease. Recurrence is defined after a previous GPA diagnosis followed by months of disease-free. Follow-up was measured in months from the time of primary treatment until death or last contact.

## 3. Results

### 3.1. Main Findings

A total of 1422 references (1199 Pubmed + 195 Embase + 25 Free terms + 3 hand searching) were identified from electronic databases in the search performed on 21 July 2021. One thousand three hundred and seventy-three citations were excluded based on the title and/or abstract, duplicate entries, and written in languages other than English, thereby 49 potentially relevant articles were included for subsequent evaluation. Most of these studies were excluded due to pregnancy (11), fertility (11), or related to other aspects of gynecology (5).

The pathological diagnosis of vasculitis in patients with GPA, microscopic polyangiitis (MPA), or eosinophilic granulomatosis with polyangiitis (EGPA, Chug-Strauss syndrome) is difficult, sometimes not possible. We excluded five patients with genital tract involvement from our review, one with MPA and four with EGPA [7,8,9,10,11]. 

**Figure 1:**
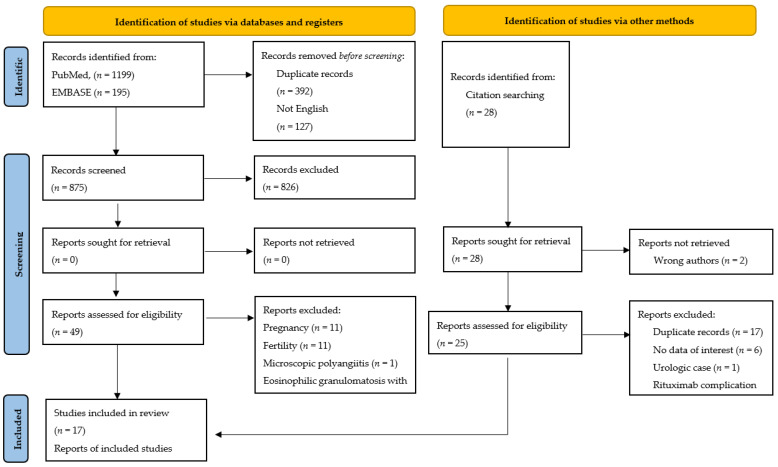
Flow diagram of study search and systematic review.

Once studies with patients with MPA or EGPA (5) were removed, a total of 17 studies fulfilled all inclusion criteria and were selected for full-text evaluation and systematic review [12,13,14,15,16,17,18,19,20,21,22,23,24,25,26,27,28]. After reviewing the 17 articles, 18 patients were identified as GPA with gynecological involvement (two patients reported by Stone et al.) [12]. A flow diagram of the study search is shown in Figure 1.

A total of nineteen patients, including the present case, constitute the basis of the systematic review. Among them, 11 patients (58%) had primary gynecologic involvement, and 8 had genital recurrences (42%). In the primary GPA group, one patient had a cutaneous recurrence at 12 months and is included as recurrence [23].

The median age of the cohort was 55 years (range 32–82), and the median diagnostic delay was 12 months (range 0–60). Initial presentation of primary GPA with exclusively gynecologic symptoms occurred in only two patients (18%). The main gynecological symptom was genital bleeding (16/19, 84%). The coexistence of symptoms in other organs was as follows: ERN symptoms (12/19, 63%), pulmonary (8/19, 44%), and urologic (10/19, 53%). The results are shown in Table 1 and Table 2.

Isolated genital involvement and multi-site involvement were present in 10 and in 9 out of 19 patients, respectively (43%). The most affected areas of the genital tract were the uterine cervix (15/19, 79%) and the vagina (8/19, 42%). The distribution of affected areas was as follows: cervix (7), cervix-vaginal (6), vagina (1), vulva (1), ovary (1), cervix-ovary (1), cervix-uterus (1), and vagina-urethra (1). The clinical presentation included a mass (8), granuloma, nodule, or ulcer (3 respectively), and necrosis (2).

In thirteen of the 19 patients (68%) [14,15,16,17,18,20,21,22,24,25,26,27,28], including our case, the authors considered gynecologic cancer as the first possibility of diagnosis. Among them, 11 (58%) thought they were dealing with cervical cancer [14,15,16,17,18,21,22,24,25,26,27,28].

### 3.2. Therapy for GPA

The most commonly used regimen in induction therapy was glucocorticoids + cyclophosphamide (9 of 14, 64%). Data on treatment efficacy were obtained from 12 patients, CR was achieved in 9 patients (75%), six of them treated with glucocorticoid + cyclophosphamide regimen. Maintenance therapy was available from 14 patients; the most commonly used regimens were glucocorticoids + cyclophosphamide (4), glucocorticoids + methotrexate (4), and glucocorticoids + azathioprine (3). There were 6/8 CR (75%), and all regimens used had similar results. Rituximab was used for primary and recurrent disease in five studies for induction and maintenance therapy. CR was obtained in all patients with primary disease, including our patient. The results of the different treatments are summarized in Table 3 and Table 4.

### 3.3. Outcomes

The median follow-up of the cohort was 32.5 months (range 1–180). The median time to the first recurrence was 36 months (range 10–180 months), and the median time to second recurrence was 40 months (range 18–96 months). The mortality rate was 16% (3/19). Two patients died within 43 days from diagnosis and the third one within 5 years. The cause of death was unknown. This section may be divided into subheadings. It should provide a concise and precise description of the experimental results, their interpretation, as well as the experimental conclusions that can be drawn.

## 4. Discussion

Our systematic review identified 19 patients with GPA and genital tract involvement [12,13,14,15,16,17,18,19,20,21,22,23,24,25,26,27,28]. Among them 11/19 (58%) had primary disease, and 8/19 (42%) had recurrence. A primary diagnosis of GPA is more difficult when the genital tract is the first and only manifestation of the disease, especially when the pathological examination is inconclusive, such as in our patient. The diagnosis is usually, but now always, easier with a previous history of GPA elsewhere in the body [12,21,22,23,24,25,26,27,28].

The most common clinical presentation of primary GPA is that of a gynecologic malignancy, as noted in 68% of the studies reviewed [2,16,17,18,19,22,23,24,26,27,28,29,30]. Some of these patients received incorrect treatments such as surgical excision, anti-tuberculosis treatment, and/or radiotherapy [13,14,15,18,20,21,24,28].

Most patients have additional symptomatology in addition to genital symptoms (ENT (63%), lungs (44%), kidneys-urinary tract (53%)), exclusive gynecologic symptomatology is rare and only noted in 18% of patients [14,22].

Isolated cervical involvement in GPA is uncommon (less than 1%)^5^ and in our systematic review was observed in seven patients. Cervical disease usually develops as a late manifestation after a mean interval of 12 years and is commonly (79%) associated with vaginal disease (Table 1 and Table 2). The most common symptom is bleeding (84%), and the most common diagnosis is cervical cancer due to the presence of necrosis or a necrotic mass that bleeds on slight touch (Table 1 and Table 2).

Our patient presented with vaginal bleeding and intermittent hematuria and was thought to have advanced vaginal cancer. A diagnosis of GPA was suspected but not established until she responded to a short therapeutic trial of rituximab. Usually, histological analysis of biopsies from affected organs confirms the diagnosis, showing necrotizing vasculitis and granulomatous inflammation. Its hallmark features include necrotizing granulomatous inflammation and pauci-immune vasculitis in small- and medium-sized blood vessels. Although tissue for diagnosis and/or prognosis is usually obtained from a skin or kidney biopsy, histological lesions of the airways and lung parenchyma are more cost-effective and characterized by intravascular and extravascular necrotizing granulomas with abundant multinucleated giant cells and necrotizing vasculitis involving small arteries and veins. In the present case, inconclusive biopsies and blood test results, the absence of other organ involvement, and the existence of only one criterion—intermittent hematuria—rather than the minimum of two criteria required for a clinical GPA diagnosis created a diagnostic challenge. Necrotizing deep tissue infections (e.g., mycobacterial, fungal) and other vasculitides have similar histologic features but without necrotizing vasculitis. After infectious processes and other vasculitides were ruled out, a diagnosis of GPA was considered. Consultation with the rheumatology service was then obtained.

In the systematic review, ANCA testing plays a critical role in the diagnosis and classification of vasculitides, even as debate about their ultimate importance in the pathogenesis and pathophysiology of these conditions continues. Two types of antineutrophil cytoplasmic autoantibody (ANCA) assays are in wide use: (a) Indirect immunofluorescence assay, using alcohol-fixed buffy coat leukocytes; (b) Enzyme-linked immunosorbent assay (ELISA), using purified specific antigens. There are several types of ANCA antibodies depending on the specific protein to which they bind to CD20, cANCA to proteinase 3 (PR3), and pANCA to myeloperoxidase (MPO). The latter are the ones that could be found in another vasculitis than GPA; however, the presence of cANCA is very specific (95%) and sensitive (88%) for GPA. In more than 95% of patients with active disease, cANCA is detected.

According to the current literature, an induction regimen consisting of glucocorticoids in combination with rituximab or cyclophosphamide is recommended. Following CR, rituximab has been the maintenance therapy of choice [17] since 2009, when it was first used [26].

Significant morbidity results from the disease and its treatment, such end-stage renal disease and adrenal or multiorgan failure [12,19,20,21,24,26,29]. As indicate above the mortality rate of gynecological disease is 16% [15,21,24], as compared to slightly higher than 25% [2]. A higher recurrence rate is observed with a diagnosis delay of 12 months or longer. However, there is not a single patient with genital involvement and without a uterus (previous hysterectomy or performed at diagnosis) who has experienced a recurrence, including our patient [14,15,18,19].

The main limitations of this review are related to the high heterogeneity of the published data, the lack of definitions in the manuscripts reviewed (delay in diagnosis, recurrence, follow-up), incomplete outcome data (CR or PR) and loss of follow-up, variability in the diagnostic approach and in the medication used during the therapeutic regimen followed (induction and/or maintenance). In addition, we are aware of the loss of two cases (vulva and cervix) as they are written in a language other than English [30,31]. In this context, we consider that a meta-analytic approach is not feasible and could even contain biases. The main strength of our study is that it provides a better understanding of what is already known about patients with GPA and gynecologic involvement, suggesting some clues for diagnosis and medical decision making but also pointing out the need for future research. Future research should take into account the limitations described above.

## 5. Conclusions

GPA is a rare chronic condition that can infrequently affect the female genital tract. A diagnosis of GPA must be considered when biopsies of a malignant-appearing cervical or vaginal necrotic or ulcerated mass are negative for malignancy, even with the unspecific pathological examination, with or without other organ involvement. Treatment for GPA is effective.

## Figures and Tables

**Table 1:** Literature review of patients with GPA and primary gynecological involvement.

Authors	Age, yr	Clinical Evolution	Initial Presentation of Symptoms	ORL Symptoms	Lung Findings	Urological Symptoms	Gynecologic Symptoms	Examination Findings	Diagnosis Delay
Stone et al., 1997 [12]	55	First symptoms: mild-1970s First consultation: 1982 Diagnosis: 1996	Saddle nose and upper respiratory tract	Yes	No	No	Vaginal bleeding	Nodular lesions in cervix and vagina	21 yr
Hendrix et al., 2009 [13]	82	Four yr-history of symptoms First consultation and diagnosis at the same time after 4 yr	Vaginal blood loss. Cervix then lung	No	Yes	No	Vaginal bleeding and purulent dischage	Cervical necrotizing granulomatous inflammation and vaginal ulcer	48 mo ^+^
Oladipo et al., 2011 [14]	71	1 yr-history of symptoms Diagnosis was made 2 months after vault granuloma excision	Postmenopausal bleeding	No	No	No	Postmenopausal bleeding	Exophytic growth on the cervix with contact bleeding	12 mo ^+^
Nan et al., 2014 [15]	58	First symptoms: September 2012 On day 30: GPA suspected On day 37: Diagnosis.	Abnormal vaginal bleeding and compromise of multiple organs and systems	Yes	Yes	Yes	Vaginal bleeding	Partial hysterectomy, 9 yr before. Nodular lesions in cervix and ovarian cysts with purulent spillage	37 d
Caron et al., 2014 [16]	48	One yr-history of symptoms First consultation and diagnosis at the same time after 1 yr	Abnormal cervix and ENT	Yes	Yes	No	Bleeding after sexual intercourse	Soft and friable mass that replaced the cervix	12 mo
Campochiaro et al., 2016 [17]	55	Two mo-history of symptoms Diagnosis was made two weeks later of initial symptoms	Exophytic cervical lesion then synovitis, purpura, and red papules	No	Yes	Yes	Metrorrhagia	Necrotic cervical mass extending to the lower third vaginal wall.	2 mo (First work up ^+^)
Fallahi et al., 2017 [18]	33	Two mo-history of symptoms First consultation: May 2015 Diagnosis was made during admission	Lower abdominal pain, and abnormal uterine bleeding	Yes	Yes	Yes	Abnormal uterine bleeding	Uterine mass and cervical necrotizing vasculitis	2 mo ^+^
Soro et al., 2017 [19]	62	Three mo-history of symptoms Diagnosis was made during admission	Vesical tenesmus and vaginal bleeding and low-grade fever	No	No	Yes	Vaginal bleeding	Hysterectomy, BSO 12 yr before. Irregular hardening of anterior vagina encompassing urethra	3 mo
Bielejewska et al., 2020 [20]	48	Diagnosis was made during hospitalization	Lumbosacral pain, left hydronephrosis, then nasal ulcer	Yes	No	Yes	Pelvic pain	Necrotizing and granulomatous inflammatory tumour left ovary	No
Pereira et al., (present case)	75	One-year history of symptoms	Pelvic pain, intermittent hematuria, vaginal bleeding, and pelvic mass	No	No	Yes	Pelvic pain and postmenopausal bleeding	Hysterectomy, BSO 36 yr before. Ulcerative, exophytic vaginal mass with contact bleeding	12 mo

Abbreviation: yr: years; GPA: granulomatosis polyangiitis; ENT: Ear, nose and throat; mo: months; wk: week; BSO: Bilateral salpingo-oophorectomy; +: Published data by other author.

**Table 2:** Literature review of patients with previous history of GPA and gynecological relapse.

Authors	Age, yr	Clinical Evolution	Initial Presentation of Symptoms	ORL Symptoms	Lung Findings	Urological Symptoms	Gynecologic Symptoms	Examination Findings	Diagnosis Delay
Fridman et al., 1964 [21]	61	First consultation: July 1958 Diagnosis: November 1960 First recurrence: September 1961 Second recurrence: 1962	ENT	Yes	No	No	Gynecological complaint	Uterine cervix and vaginal granuloma	28 mo (Undisclosed ^+^)
Stone et al., 1997 [12]	67	First symptoms: 1988 Diagnosis: 1993 Recurrence: 1995	Nose and lungs	Yes	Yes	Yes	Vaginal discharge	Nodular lesions on the cervix and vaginal wall	60 mo
Malamou-Mitsi et al., 2000 [22]	45	Fifteen-year history of GPA Diagnosis of recurrence was made during admission	Unspecified	No	No	No	Vaginal bleeding	Necrotic and easily bleeding ectocervix mass with abnormal vessels	Undisclosed ^+^
Lewis et al., 2002 [23]	54	The diagnosis was made after 2 wk-history of symptoms. Recurrence 1 yr later	Iritis left eye. Deep ulceration of the heart palate then vulva	Yes	No	No	Vulvar soreness and contact bleeding	Vasculitic lesions on both labia minora	<2 wk
Ahson et al., 2002 [24]	80	Three-year history of GPA Diagnosis of recurrence was made after 10 mo-history of symptoms	Kidneys, end-stage renal disease	No	No	Yes	Postmenopausal bleeding	Cervical necrosis and contact bleeding	16 mo ^+^
Bean and Conner 2007 [25]	32	Past medical history of GPA Diagnosis: 1999 One yr-history of symptoms before recurrence	Sinuses, nose, lungs	Yes	Yes	Yes	Vaginal bleeding	Cervical necrosis andcontact bleeding	12 mo ^+^
Maina et al., 2009 [26]	64	Ten-year history of GPA 2004: first recurrence January 2008: Diagnosis of second recurrence was made after 3 wk-history of symptoms:	Ear, eye, and renal involvement	Yes	No	Yes	Postmenopausal bleeding	Highly vascular and irregular cervical mass involving 50% of ectocervix	3 wk ^+^
Mukherjee et al., 2011 [27]	42	First consultation: 2003 Diagnosis: 2005 First recurrence: March 2008 Second recurrence: May 2008? persist	ENT and irregular and inflamed cervix	Yes	No	No	Intermenstrual and postcoital bleeding	Large friable vascular area and necrotizing granulomata in cervix	24 mo (5 yr ^+^)
Bastone et al., 2015 [28]	34	Diagnosis: March 2008 First recurrence: March 2011 Second recurrence: July 2014	Skin, joints, sinus	Yes	No	No	Metrorrhagia	Uterine cervix and upper vagina	19 mo ^+^

Abbreviation: yr: years; GPA: granulomatosis polyangiitis; ENT: Ear, nose, and throat; mo: months; wk: week; BSO: Bilateral salpingo-oophorectomy; +: Published data by another author.

**Table 3:** Literature review of patients with GPA and primary gynecological involvement.

Authors	Treatment	Outcome	Time	First Recurrence	Interval	Second Recurrence	Interval	Patient Status	Follow-Up
Stone et al., 1997 [12]	No treatment until diagnosis in 1996: Cyclophosphamide and prednisone *	CR *	1 mo	-	-	-	-	Alive	1 mo *
Hendrix et al., 2009 [13]	Treatment from diagnosis: Cyclophosphamide and prednisone *	Fast clinical recovery *	-	-	-	-	-	Alive	-
Oladipo et al., 2011 [14]	After 1 yr-history of symptoms Hysterectomy Azathioprine *	CR *	6 mo	-	-	-	-	Alive	12 mo *
Nan et al., 2014 [15]	On day 30: Methylprednisolone On day 39: 2. Cyclophosphamide and methylprednisolone *	The patient fell into a coma and died the following day	43 d	-	-	-	-	Died Unknown cause	44 d
Caron et al., 2014 [16]	Treatment started without delay: Methotrexate and prednisone *	CR *	1 mo	-	-	-	-	Alive	1 mo *
Campochiaro et al., 2016 [17]	After 2-mo history of symptoms: Methylprednisolone * Cyclophosphamide and prednisone * One month later: 3. Rituximab *	CR *	3 mo	-	-	-	-	Alive	3 mo *
Fallahi et al., 2017 [18]	After first consultation: May 2015. Hysterectomy Treatment started 2 months after initial symptoms: 2. Cyclophosphamide and prednisolone *	PR *	6 mo	-	-	-	-	Alive	6 mo *
Soro et al., 2017 [19]	After 3 mo-history of symptoms Prednisone and methotrexate * Two yr later: 2. Prednisone and azathioprine ** Four yr later: 3. Prednisone and methotrexate ***	Sustained remission *	-	-	-	-	-	Alive	60 mo
		Vagino-urethral fistula **	2 yr **						
		CR ***	5 yr ***						
Bielejewska et al., 2020 [20]	Treatment started during first hospitalization: Removal of left uterine appendages * Treatment after progression: 2. Cyclophosphamide and methylprednisone ** 3. Oral prednisone ** Treatment during re-hospitalization: High dose of cyclophosphamide ***	Progression of GPA *	Later	-	-	-	-	Alive	12 mo
		Improvement without recovering renal function **	-						
		General good condition ***	-						
		Kidney transplant	1 yr						
Pereira et al. (present case)	After 1-year history of symptoms Rituximab and prednisone * Four wk later: 2. Methotrexate and prednisone *	CR *	6 mo	-	-	-	-	Alive	53 mo

Abbreviation: yr: years; GPA: granulomatosis polyangiitis; mo: month; wk: week; CR: Complete remission; PR: Partial remission; *: First treatment; **: Second treatment; ***: Third treatment.

**Table 4:** Literature review of patients with previous history of GPA and gynecological involvement.

Authors	Treatment	Outcome	Time	First Recurrence	Interval	Second Recurrence	Interval	Patient Status	Follow-Up
Fridman et al., 1964 [21]	No treatment until 1962. Treatment since recurrence: Radiotherapy Cortisone *	Dramatic improvement *	-	Turbinate	10 mo	Cervix-vagina	18 mo	Died Unknown cause	Treatment: 1 mo * Total:5 yr
Stone et al., 1997 [12]	First treatment: 1988 Corticosteroid therapy Treatment from diagnosis 1993: 2. Cyclophosphamide and methylprednisolone * 3. Methotrexate and prednisone ** Treatment since recurrence: 4. Cyclophosphamide and prednisone ***	CR * Well ** CR ***	16 mo * 13 mo ** 2 wk ***	Vulva Urethra	24 mo	-	-	Alive	Treatment: 38 mo - 16 mo * - 13 mo ** - 9 mo *** Total: 7 yr
Malamou-Mitsi al., 2000 [22]	15-yr history of GPA treated with cyclophosphamide Treatment since recurrence: Cyclophosphamide and prednisone *	CR *	3 mo *	Cervix	180 mo	-	-	Alive	Treatment: 12 mo Total: 15 yr
Lewis et al., 2002 [23]	Diagnosis after <1 mo of initial symptoms Prednisolone * Treatment since recurrence: 2. Prednisolone added azathioprine **	Resolved *	Rapidly *	Skin	12 mo	-	-	Alive	Treatment: 12 mo * - Lost ** Total: 12 mo
Ahson et al., 2002 [24]	Treatment from 1989 until July 2001 Prednisolone and azathioprine *	Died 1 day after hysteroscopy	-	Cervix	36 mo	-	-	Died Unknown cause	Treatment: 12 yr * Total: 12.5 yr
Bean and Conner 2007 [25]	Treatment after diagnosis 1999: Cyclophosphamide and prednisone *	Not provided	-	Cervix-vagina	84 mo	-	-	Alive	Treatment: Lost * Total: 1 yr
Maina et al., 2009 [26]	10-yr history of GPA treated with methotrexate * Treatment first recurrence: 2004 Treatment second recurrence: 2008 Rituximab and prednisone **	Not provided*	-	Sclera	48 mo	Cervix Thraquea	96 mo	Alive	Treatment: Lost ** Total: 10 yr
Mukherjee et al., 2011 [27]	First treatment: January 2006 Cyclophosphamide and prednisolone * Azathioprine Treatment since recurrence: March 2008 3. Cyclophosphamide following Methotrexate ** 4. Rituximab and oral steroids ***	Immediate improvement *	6 mo *	Cervix	23 mo	Upper airway	25 mo	Alive	Treatment: 29 mo - 26 mo * - 3 mo ** - Lost *** Total: 5 yr
		Definitive improvement in cervix, but upper airway not controlled **	3 mo **						
Bastone et al., 2015 [28]	Treatment since March 2008 Cyclophosphamide and methylprednisolone * Methotrexate March 2011 3. Conization: 4. Methotrexate ** November 2012 5. Rituximab ***	Promptly achieved *	-	Cervix-vagina	36 mo	Cervix-vagina	55 mo	Alive	Treatment: 50 mo - 22 mo * - 20 mo ** - 6 mo *** Total: 6.3 yr
		No remission **	20 mo **						
		PR ***	6 mo ***						

Abbreviation: yr: years; GPA: granulomatosis polyangiitis; mo: month; wk: week; CR: Complete remission; PR: Partial remission; *: First treatment; **: Second treatment; ***: Third treatment.

## Data Availability

Data presented in this manuscript are available from the corresponding authors on reasonable request.
